# Supplementary material for: Simplifying the Centrolene buckleyi complex (Amphibia: Anura: Centrolenidae): a taxonomic review and description of two new species
Source: PeerJ. 2024 Aug 20;12:e17712. doi: 10.7717/peerj.17712 (PMC11348905; doi:10.7717/peerj.17712)
Supplement: Supplemental Information 3 — Model abbreviations are as follows: BAYAREALIKE = Bayesian inference of historical biogeography for many discrete areas (with likelihood interpretation); DEC = Dispersal-Extinction-Cladogenesis; DIVALIKE = Dispersal-Vicariance Analysis (with likelihood interpretation). LnL = Log-likelihood score; d = dispersal; e = extinction; AICc = standard correction to Akaike’s Information Criterion. [file peerj-12-17712-s003.docx]

| **Model** | **Ln*L*** | **d** | **e** | **AICc** | **Delta AICc** | **AICc wt** |
| --- | --- | --- | --- | --- | --- | --- |
| BAYAREALIKE | -49.7905 | 0.1092 | 0.2640 | 104.3312 | 22.8131 | 1.1E-05 |
| DEC | -42.5888 | 0.0983 | 0.0379 | 89.9276 | 8.4095 | 0.015 |
| DIVALIKE | -38.3840 | 0.0891 | 7.1341E-09 | 81.5181 | 0 | 0.99 |
