# Supplementary material for: Simplifying the Centrolene buckleyi complex (Amphibia: Anura: Centrolenidae): a taxonomic review and description of two new species
Source: PeerJ. 2024 Aug 20;12:e17712. doi: 10.7717/peerj.17712 (PMC11348905; doi:10.7717/peerj.17712)
Supplement: Supplemental Information 4 — Sequences downloaded from Genbank are identified by their codes and sequences generated in this study are in bold. [file peerj-12-17712-s004.docx]

| **Species** | **Museum Number** | **12S** | **16S** | **Locality** | **Latitude** | **Longitude** | **Elevation (m)** |
| --- | --- | --- | --- | --- | --- | --- | --- |
| *Celsiella vozmedianoi* | MHNLS 17877 | EU663385 | EU663025 | Venezuela: Estado Sucre, Cerro Humo. | 10.7°N | 62.61666°W | 800 |
| *Celsiella revocata* | MHNLSF 17319 | EU663379 | EU663019 | Venezuela: Estado Aragua, Colonia Tovar. | 10.4044 °N | 67.285°W | 1800 |
| *Hyalinobatrachium aureoguttatum* | QCAZ 32105 | EU663391 | EU663032 | Ecuador: Provincia Esmeraldas, on the road San Francisco–Durango. | 1.08583°N | 78.6905°W | 63 |
| *Hyalinobatrachium fleischmanni* | QCAZ 22303 | EU663405 | EU663044 | Ecuador: Provincia Esmeraldas, La Tola. | 0.40416°N | 79.911°W | 31 |
| *Hyalinobatrachium yaku* | MZUTI 5002 | MF002068 | MF002066 | Ecuador: Provincia Pastaza, stream affluent of the Kallana river. | 1.4696°S | 77.2784°W | 325 |
| *Nymphargus anomalus* | QCAZ 45702 | MH746585 | MH746559 | Ecuador: Provincia Pastaza, Río Challuwa Yaku. | 1.26764°S | 78.04797°W | 1668 |
| *Nymphargus bejaranoi* | CBG 1488 | EU663422 | EU663059 | Bolivia: Departamento Cochabamba, Chaquisacha. | 17.6833°S | 65.416°W | 1500 |
| *Nymphargus cariticommatus* | MZUTI 1417 | MH746580 | MH746554 | Ecuador: Provincia Morona Santiago, Entre Plan de Milagro y Gualaceo. | 3.00774°S | 78.53318°W | 2159 |
| *Nymphargus cochranae* | QCAZ 31113 | EU663425 | EU663061 | Ecuador: Provincia Napo, Pacto Sumaco. | 0.716666°S | 77.5666°W | 1400 |
| *Nymphargus garciae* | KU 20801 | AY326022 | AY326022 | Ecuador: Provincia Sucumbíos, 18 km E Santa Bárbara. | – | – | 2550 |
| *Nymphargus griffithsi* | MZUTI 100 | MH830303 | MH830299 | Ecuador: Provincia Pichincha, Reserva Las Gralarias (). | 0.031111°S | 78.70666°S | 2175 |
| *Nymphargus lasgralarias* | MZUTI 096 | MH746569 | MH746544 | Ecuador: Provincia Pichincha, Reserva Las Gralarias. | 0.04083°S | 78.7097°W | 2150 |
| *Nymphargus lindae* | QCAZ 41572 | MH746582 | MH746556 | Ecuador: Provincia Zamora Chinchipe, Miazi Alto. | 4.25044°S | 78.61356°W | 1200 |
| *Nymphargus megacheirus* | KU 143272 | EU663427 | EU663063 | Ecuador: Provincia Napo, 16.5 km NE Santa Rosa. | 0.2166°S | 77.71666°W | 1700 |
| *Nymphargus rosada* | MHUA 4308 | EU663429 | EU663066 | Colombia: Departamento Antioquia, Finca El Chaquiral. | 0.658°N | 75.783°W | 1732 |
| *Nymphargus siren* | KU 179171 | EU663430 | EU663067 | Ecuador: Provincia Napo, 3.2 km NE Oritoyacu. | 0.45°N | 77.866°W | 1910 m |
| *Nymphargus sucre* | MZUTI 1421 | MH746578 | MH746552 | Ecuador: Provincia Morona Santiago, Camino entre Plan de Milagro y Gualaceo. | 3.00774°S | 78.53318°W | 2159 |
| *Nymphargus vicenteruedai* | AVV 119 | EU663424 | EU663058 | Colombia: Departamento Santander, Río Cercados. | – | – | 2650 |
| *Nymphargus wileyi* | QCAZ 27435 | EU663431 | EU663068 | Ecuador: Provincia Napo, Yanayacu Biological Station. | 1.72553°S | 78.98058°W | 2100 |
| *Centrolene altitudinalis* | MHNLS 17225 | EU663334 | EU662975 | Venezuela: Estado Mérida: Quebrada Albarregas. | 8.6166°N | 71.15°W | 2100 |
| *Centrolene altitudinalis* | MHNLS 17194 | EU663333 | EU662974 | Venezuela: Estado Mérida: Quebrada Albarregas. | 8.6166°N | 71.15°W | 2100 |
| *Centrolene antioquiense* | NRPS 014 | EU663336 | EU662977 | Colombia: Departamento Antioquia, bosque de la Forzosa. | – | – | 2127 |
| *Centrolene* aff. *buckleyi* | MAR 371 | EU663339 | EU662980 | Colombia: Departamento Cundinamarca: Parque Nacional Chingaza. | – | – | 3035 |
| *Centrolene* aff. *condor* | QCAZ 44896 | KF639755 | JX126955 | Ecuador: Provincia Zamora Chinchipe, Los Encuentros. | – | – | – |
| *Centrolene* aff. *savagei* | MAR 1152 | KM068295 | KM068295 | Colombia: Departamento del Chocó, Estribaciones del Cerro Tacarcuna. | – | – | 260 |
| *Centrolene ballux* | QCAZ 40186 | KF639754 | HG764783 | Ecuador: Provincia Pichincha, Reserva las Gralarias. | – | – | 2075 |
| *Centrolene buckleyi* | MZUTI 763 | MH844843 | MH844849 | Ecuador: Provincia Napo: Oyacachi. | 0.2189°S | 78.044°W | 3012 |
| *Centrolene buckleyi* | KU1 78031 | EU663338 | EU662979 | Ecuador: Provincia Imbabura, Near Lago Cuicocha. | 0.3025°S | 78.618611°W | 3010 |
| *Centrolene buckleyi* | DHMECN 867 | **‒** | **0R479083** | Ecuador: Provincia Bolívar, Guanujo. | 1.555557°S | 79.006666°W | 2900 |
| *Centrolene buckleyi* | DHMECN 13828 | **OR479108** | **0R479085** | Ecuador: Provincia Carchi, San Francisco de Pioter. | 0.67113888°N | 77.79552777°W | 3416 |
| *Centrolene buckleyi* | DHMECN 14180 | **‒** | **0R479086** | Ecuador: Provincia Carchi, Maldonado. | 0.767882°N | 78.056111°W | 2841 |
| *Centrolene buckleyi* | DHMECN 13376 | **‒** | **‒** | Ecuador: Provincia Carchi, Morán. | 0°46'07.10"N | 78°03'20.00"O | 2785 |
| *Centrolene buckleyi* | CJ 1055 | **OR479115** | **0R479093** | Ecuador: Provincia Cotopaxi, Sigchos. | 0.7°S | 78.88333°W | 2770 |
| *Centrolene buckleyi* | CJ 2171 | **OR479110** | **0R479088** | Ecuador: Provincia Cotopaxi, Río Unachi. | 0.68166°S | 78.09874°W | 2677 |
| *Centrolene buckleyi* | CJ 9789 | **OR479114** | **0R479092** | Ecuador: Provincia Cotopaxi, Río Unachi. | 0.68166°S | 78.09874°W | 2677 |
| *Centrolene buckleyi* | CJ 4292 | **OR479113** | **0R479091** | Ecuador: Provincia Cotopaxi, Río Unachi. | 0.68166°S | 78.09874°W | 2677 |
| *Centrolene buckleyi* | CJ 11305 | OR479111 | 0R479089 | Ecuador: Provincia Pichincha, Atahualpa. | 0.16496°N | 78.33361°W | 2573 |
| *Centrolene buckleyi* | **EPN 2418** | ‒ | ‒ | Ecuador: Provincia Bolívar, Guanujo. | 1.555557°S | 79.006666°W | 2865 |
| *Centrolene buckleyi* | **EPN 2695** | ‒ | ‒ | Ecuador: Provincia Bolívar, Guaranda. | 1.555557°S | 79.006666°W | 2865 |
| *Centrolene buckleyi* | **EPN 2702** | ‒ | ‒ | Ecuador: Provincia Imbabura, Laguna de Cuicocha. | 0.300166°N | 78.364235°W | 3062 |
| *Centrolene buckleyi* | **EPN 2703** | ‒ | ‒ | Ecuador: Provincia Imbabura, Laguna de Cuicocha. | 0.300166°N | 78.364235°W | 3062 |
| *Centrolene buckleyi* | **EPN 3209** | ‒ | ‒ | Ecuador: Provincia Imbabura, Laguna de Cuicocha. | 0.300166°N | 78.364235°W | 3062 |
| *Centrolene buckleyi* | **EPN 3210** | ‒ | ‒ | Ecuador: Provincia Imbabura, Laguna de Cuicocha. | 0.300166°N | 78.364235°W | 3062 |
| *Centrolene buckleyi* | **EPN 3211** | ‒ | ‒ | Ecuador: Provincia Imbabura, Laguna de Cuicocha. | 0.300166°N | 78.364235°W | 3062 |
| *Centrolene buckleyi* | **EPN 3212** | ‒ | ‒ | Ecuador: Provincia Imbabura, Laguna de Cuicocha. | 0.300166°N | 78.364235°W | 3062 |
| *Centrolene buckleyi* | **EPN 3213** | ‒ | ‒ | Ecuador: Provincia Imbabura, Laguna de Cuicocha. | 0.300166°N | 78.364235°W | 3062 |
| *Centrolene buckleyi* | **EPN 3214** | ‒ | ‒ | Ecuador: Provincia Imbabura, Laguna de Cuicocha. | 0.300166°N | 78.364235°W | 3062 |
| *Centrolene buckleyi* | **EPN 3215** | ‒ | ‒ | Ecuador: Provincia Imbabura, Laguna de Cuicocha. | 0.300166°N | 78.364235°W | 3062 |
| *Centrolene buckleyi* | **EPN 3216** | ‒ | ‒ | Ecuador: Provincia Imbabura, Laguna de Cuicocha. | 0.300166°N | 78.364235°W | 3062 |
| *Centrolene buckleyi* | **EPN 3217** | ‒ | ‒ | Ecuador: Provincia Imbabura, Laguna de Cuicocha. | 0.300166°N | 78.364235°W | 3062 |
| *Centrolene buckleyi* | **EPN 3218** | ‒ | ‒ | Ecuador: Provincia Imbabura, Laguna de Cuicocha. | 0.300166°N | 78.364235°W | 3062 |
| *Centrolene buckleyi* | **EPN 3219** | ‒ | ‒ | Ecuador: Provincia Imbabura, Laguna de Cuicocha. | 0.300166°N | 78.364235°W | 3062 |
| *Centrolene buckleyi* | **EPN 3521** | ‒ | ‒ | Ecuador: Provincia Imbabura, Laguna de Cuicocha. | 0.300166°N | 78.364235°W | 3062 |
| *Centrolene buckleyi* | **EPN 3522** | ‒ | ‒ | Ecuador: Provincia Cañar, Ingapirca. | 2.534016°S | 78.860759°W | 3361 |
| *Centrolene buckleyi* | **EPN 3592** | ‒ | ‒ | Ecuador: Provincia Bolívar, Guaranda. | 1.555557°S | 79.006666°W | 2865 |
| *Centrolene buckleyi* | **EPN 8926** | ‒ | ‒ | Ecuador: Provincia Pichincha, Machachi. | 0.493783°S | 78.525723°W | 2912 |
| *Centrolene buckleyi* | **EPN 8927** | ‒ | ‒ | Ecuador: Provincia Pichincha, Machachi. | 0.493783°S | 78.525723°W | 2912 |
| *Centrolene buckleyi* | **EPN 8928** | ‒ | ‒ | Ecuador: Provincia Pichincha, Machachi (0.493783°S, 78.525723°W; 2912 m). | 0.493783°S | 78.525723°W | 2912 |
| *Centrolene buckleyi* | **ZSFQ 4420** | **OR479107** | **0R479084** | Ecuador: Provincia Carchi, Loma La Esperanza. | 0.768527777°N | 78.0555833°W | 2934 |
| *Centrolene buckleyi* | **ZSFQ 4421** | **OR479109** | **0R479087** | Ecuador: Provincia Carchi, Morán. | 0.768333333°N | 78.051861111°W | 2784 |
| *Centrolene buckleyi* | **ZSFQ 5366** | **OR479112** | **0R479090** | Ecuador: Provincia Cotopaxi, Angamarca. | 1.13628°S | 78.89587°W | 3063 |
| *Centrolene camposi* | DHMECN 11407 | OQ225629 | OQ225616 | Ecuador: Provincia Azuay, La Enramada. | 3.1628°S | 79.5886°W | 2950 |
| *Centrolene charapita* | MHNC 13933 | KM068247 | KM068256 | Peru: Departamento Amazonas, La Oliva. | 5.30107°S | 78.3957°W | 682 |
| *Centrolene charapita* | MNCN45392_JMG2013 | KF639760 | KF534358 | Peru: Departamento Amazonas, before La Oliva. | 5.301083°S | 78.39569°W | 664 |
| *Centrolene condor* | QCAZ 72514 | ‒ | OQ225617 | Ecuador: Provincia Morona Santiago, Reserva Biologica El Quimi | – | – | – |
| *Centrolene* cf. *elisae* | ZSFQ 2134 | **OR479116** | **0R479098** | Ecuador: Provincia Sucumbios, Santa Barbara. | 0.65036°N | 77.50072°W | 2671 |
| *Centrolene* cf. *venezuelense* | IAvH-Am-17401 | **OR479122** | **0R479100** | Colombia: Departamento Cundimarca, Tocancipá. | 4.95609°N | 73.89272°W | 2776 |
| *Centrolene* cf. *venezuelense* | IAvH-Am-17403 | **OR479124** | **0R479102** | Colombia: Departamento Cundimarca, Tocancipá. | 4.95609°N | 73.89272°W | 2776 |
| *Centrolene* cf. *venezuelense* | IAvH-Am-17407 | **OR479125** | **0R479103** | Colombia: Departamento Cundimarca, Tocancipá. | 4.95609°N | 73.89272°W | 2776 |
| *Centrolene* cf. *venezuelense* | IAvH-Am-17410 | **OR479123** | **0R479101** | Colombia: Departamento Cundimarca, Tocancipá. | 4.95474°N | 73.89253°W | 2795 |
| *Centrolene daidalea* | MHUA 3271 | EU663366 | EU663007 | Colombia: Departamento Cesar, San Cayetano. | 8.425027°N | 73.4009°W | 1600 |
| *Centrolene elisae* sp. nov. | MZUTI 83 | MH844840 | MH844846 | Ecuador: Provincia Napo: Las Caucheras. | 0.61332°S | 77.89741°W | 2187-2190 |
| *Centrolene elisae* sp. nov. | MZUTI 84 | MH844841 | MH844847 | Ecuador: Provincia Napo: Las Caucheras. | 0.61332°S | 77.89741°W | 2187-2190 |
| *Centrolene elisae* sp. nov. | MZUTI 85 | MH844842 | MH844848 | Ecuador: Provincia Napo: Las Caucheras. | 0.61332°S | 77.89741°W | 2187-2190 |
| *Centrolene elisae* sp. nov. | QCAZ 25744 | MT225170 | ‒ | Ecuador: Provincia Napo: Yanayacu Biological Station. | 0.599202°S | 77.88965°W | 2100 |
| *Centrolene elisae* sp. nov. | ZSFQ 4228 | OR479117 | 0R479099 | Ecuador: Provincia Tungurahua, Reserva Chamana. | 1.4237271°S | 78.393167°W | 2586 |
| *Centrolene elisae* sp. nov. | **ZSFQ 5367** | ‒ | ‒ | Ecuador: Provincia Napo, Yanayacu Biological Station. | 0.61427°S | 77.88214°W | 2116 |
| *Centrolene elisae* sp. nov. | **ZSFQ 5368** | ‒ | ‒ | Ecuador: Provincia Napo, Yanayacu Biological Station. | 0.61424°S | 77.88217°W | 2118 |
| *Centrolene elisae* sp. nov. | **ZSFQ 5369** | ‒ | ‒ | Ecuador: Provincia Napo, Yanayacu Biological Station. | 0.6172583°S | 77.884125°W | 2117 |
| *Centrolene ericsmithi* | DHMECN 11406 | OQ225628 | ‒ | Ecuador: Provincia Azuay, La Enramada. | 3.1628°S | 79.5886° | 2950 |
| *Centrolene geckoidea* | KU 178015 | EU663341 | EU662982 | Ecuador: Provincia Pichincha, 1 km SW San Ignacio. | 0.44861°S | 78.7477°W | 1920 |
| *Centrolene heloderma* | QCAZ 40200 | KF639757 | JX126956 | Ecuador: Provincia Pichincha: Reserva las Gralarias (2100 m). | – | – | – |
| *Centrolene hesperia* | MHNSM 25802 | EU663345 | EU662986 | Peru: Departamento Cajamarca, Quebrada Chorro Blanco. | 6.84694°S | 79.0870°W | 1795 |
| *Centrolene huilense* | QCAZ 37230 | ‒ | JX126959 | Ecuador: Provincia Napo, Yanayacu Biological Station. | 1.72553°S | 78.98058°W | 2100 |
| *Centrolene hybrida* | MAR 347 | EU663346 | EU662987 | Colombia: Departamento Boyacá, Quebrada Las Palmitas. | – | – | 2000 |
| *Centrolene lynchi* | QCAZ 40191 | ‒ | JX126957 | Ecuador: Provincia Pichincha, Reserva las Gralarias, Río Lucy. | – | – | 1800 |
| *Centrolene lynchi* | QCAZ 40192 | KF639758 | ‒ | Ecuador: Provincia Pichincha: Reserva las Gralarias (1800 m). | – | – | – |
| *Centrolene marcoreyesi* sp. nov. | CJ 11364 | **OR479121** | **0R479097** | Ecuador: Provincia Zamora Chinchipe, Guarumales. | 3.9404828°S | 78.98689188°W | 2070 |
| *Centrolene marcoreyesi* sp. nov. | CJ 11564 | **OR479118** | **0R479094** | Ecuador: Provincia Zamora Chinchipe, Guarumales. | 3.93491°S | 78.98689188°W | 2070 |
| *Centrolene marcoreyesi* sp. nov. | CJ 12631 | **OR479120** | **0R479096** | Ecuador: Provincia Zamora Chinchipe, Guarumales. | 3.93826°S | 79.00525°W | 2109 |
| *Centrolene marcoreyesi* sp. nov. | MUTPL -A 00271 | **OR479119** | **0R479095** | Ecuador: Provincia Zamora Chinchipe, Abra de Zamora. | 3.9689°S | 79.1110°W | 2,190 |
| *Centrolene marcoreyesi* sp. nov. | ZSFQ 4417 | MH844838 | MH844844 | Ecuador: Provincia Zamora Chinchipe province, Estación Científica San Francisco. | 3.971667°S | 79.079167°W | 1,840 |
| *Centrolene marcoreyesi* sp. nov. | ZSFQ 4418 | MH844839 | MH844845 | Ecuador: Provincia Zamora Chinchipe province, Estación Científica San Francisco. | 3.971667°S | 79.079167°W | 1,840 |
| *Centrolene muelleri PV* | PV | ‒ | JX126958 | Peru: Departamento Amazonas, Cataratas de Gokta. | 6.023191°S | 77.90972°W | 2000 |
| *Centrolene muelleri* | CORBIDI 14667 | ‒ | KM068267 | Peru: Departamento Amazonas, Puente–Vilcaniza. | 5.8145138°S | 77.835052°W | 1858 |
| *Centrolene notostictum* | MAR 510 | EU663351 | EU662992 | Colombia: Departamento Norte de Santander, Quebrada Piritama. | – | – | 1800 |
| *Centrolene peristicta* | QCAZ22312 | EU663352 | EU662993 | Ecuador: Provincia Pichincha, Mindo Biology Station. | 0.078°S | 78.73194°W | 1600 |
| *Centrolene pipilata* | KU 178154 | EU663353 | EU662994 | Ecuador: Provincia Napo, Río Salado. | 0.191666°S | 77.69972°S | 1420 |
| *Centrolene sabini* | MUSM 28018 | ‒ | JX126960 | Peru. | 13.17805°S | 71.60861°W | 2750 |
| *Centrolene sanchezi* | MUTPL 601 | OP751416 | OP751399 | Ecuador: Provincia Zamora Chinchipe, Reserva Biológica Cerro Plateado. | – | – | – |
| *Centrolene sanchezi* | QCAZ 22728 | EU663337 | EU662978 | Ecuador: Provincia Napo, Yanayacu Biological Station. | 0.599202°S | 77.88965°W | 2100 |
| *Centrolene savagei* | MHUA 4094 | EU663380 | EU663020 | Colombia: Departamento Antioquia, Finca El Chaquiral. | 6.966666°N | 76.38333°W | 1732 |
| *Centrolene* sp. | ZSFQ 621 | **OR479128** | **0R479106** | Ecuador: Provincia Napo, Parque Nacional Llanganates. | 1.35282°S | 78.06319°W | 1404 |
| *Centrolene* sp. | ZSFQ 4422 | **OR479126** | **0R479104** | Ecuador: Provincia Morona Santiago, Chigüinda. | 3.2066138°S | 78.769758°W | 2373 |
| *Centrolene* sp. | ZSFQ 4423 | **OR479127** | **0R479105** | Ecuador: Provincia Morona Santiago, Chigüinda. | 3.2066138°S | 78.769758°W | 2373 |
| *Centrolene venezuelense* | MHNLS 16497 | EU663360 | EU663001 | Venezuela: Estado Mérida: Cordillera de Mérida. | – | – | – |
| *Centrolene venezuelense* | EBRG 5244 | EU663359 | EU663000 | Venezuela: Estado Mérida, Páramo de Maraisa. | 8.8419444°N | 70.731111°W | 2450 |
| *Centrolene zarza* | MUTPL 933 | OP751418 | OP751401 | Ecuador: Zamora Chinchipe, Refugio de Vida Silvestre El Zarza. | 3.8341°S | 78.5458°W | 1400–1680 |
| *Centrolene zarza* | MUTPL 932 | OP751417 | OP751400 | Ecuador: Zamora Chinchipe, Refugio de Vida Silvestre El Zarza. | 3.8341°S | 78.5458°W | 1400–1680 |
