# Supplementary figures and images for: Simplifying the Centrolene buckleyi complex (Amphibia: Anura: Centrolenidae): a taxonomic review and description of two new species
Source: PeerJ. 2024 Aug 20;12:e17712. doi: 10.7717/peerj.17712 (PMC11348905; doi:10.7717/peerj.17712)

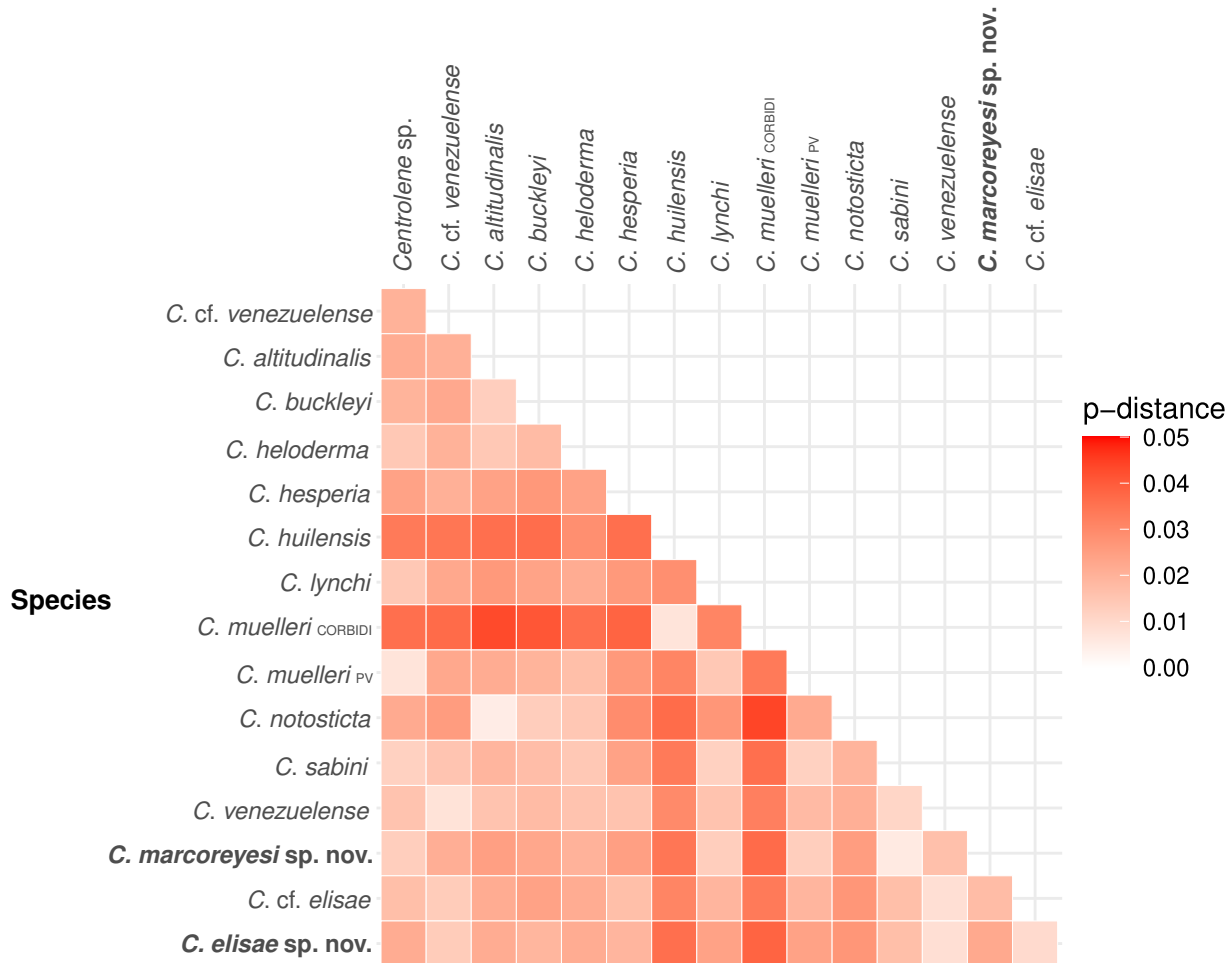

Supplement: Supplemental Information 5 [file peerj-12-17712-s005.pdf]

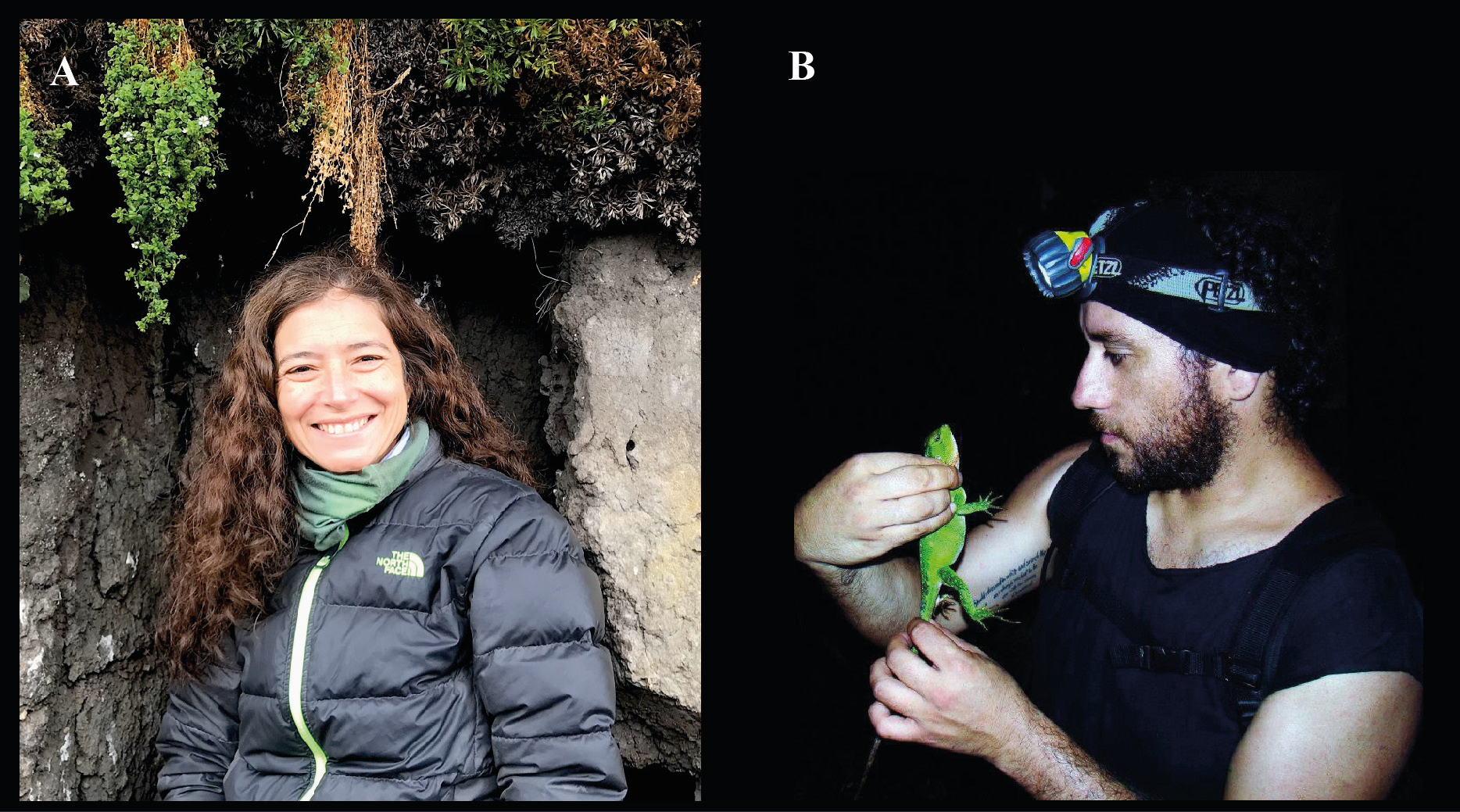

Supplement: Supplemental Information 6 [file peerj-12-17712-s006.png]
